# Effects of Malnutrition on the Immune System and Infection and the Role of Nutritional Strategies Regarding Improvements in Children’s Health Status: A Literature Review

**DOI:** 10.3390/nu16010001

**Published:** 2023-12-19

**Authors:** Fátima Morales, Sergio Montserrat-de la Paz, Maria J. Leon, Fernando Rivero-Pino

**Affiliations:** 1 Department of Preventive Medicine and Public Health, School of Medicine, University of Seville, 41009 Sevilla, Spain; fmmarin@us.es; 2 Sbarro Institute for Cancer Research and Molecular Medicine, Center of Biotechnology, College of Science and Technology, Temple University, Philadelphia, PA 19122, USA; 3 Department of Medical Biochemistry, Molecular Biology, and Immunology, School of Medicine, University of Seville, Av. Sanchez Pizjuan s/n, 41009 Seville, Spain; 4 Instituto de Biomedicina de Sevilla, IBiS, Hospital Universitario Virgen del Rocío, CSIC, University of Seville, 41013 Seville, Spain; 5 Department of Microbiology and Parasitology, School of Pharmacy, University of Seville, C. Profesor Garcia Gonzalez 2, 41012 Seville, Spain; mjl@us.es

**Keywords:** children, immunonutrition, immunometabolism, infection, nutraceuticals

## Abstract

Malnutrition refers to a person’s status as under- or overnourished, and it is usually associated with an inflammation status, which can subsequently imply a different health status, as the risk of infection is increased, along with a deterioration of the immune system. Children’s immune systems are generally more susceptible to problems than adults. In the situation of malnutrition, because malnourished children’s immune systems are compromised, they are more likely to die. However, little is known about the underlying mechanism of altered immune functioning and how it relates to starvation. Nutritional interventions have been reported as cost-effective strategies to prevent or treat the development of malnourishment, considering the link between food intake and health, especially in children, and also the susceptibility of this population to diseases and how their health status during childhood might affect their long-term physiological growth. The ingestion of specific nutrients (e.g., vitamins or oligoelements) has been reported to contribute to the proper functioning of children’s immune systems. In this review, we aim to describe the basis of malnutrition and how this is linked to the immune system, considering the role of nutrients in the modulation of the immune system and the risk of infection that can occur in these situations in children, as well as to identify nutritional interventions to improve their health.

## 1. Introduction

### 1.1. Immune System

The immune system is a complex and sophisticated network of cells, tissues, and organs that work in harmony to defend the body against a myriad of potential threats, ranging from bacteria and viruses to fungi and other pathogens. The immune system can be broadly categorized into two main components: the innate immune system and the adaptive immune system. The innate immune system provides immediate, nonspecific defense mechanisms, such as physical barriers like the skin, and cellular components like white blood cells that can rapidly respond to a wide range of pathogens. In contrast, the adaptive immune system is highly specific and can “remember” previous encounters with specific pathogens, allowing for a more targeted and efficient response upon subsequent exposures [1]. The immune system plays a crucial role in maintaining overall health and well-being. A key aspect of the immune system in the context of food is its ability to distinguish between harmless substances, like nutrients in food, and potential threats. The gut-associated lymphoid tissue is particularly important in this regard, as it is responsible for monitoring and responding to substances encountered through the digestive tract [2].

One key distinction between the immune systems of children and adults lies in their developmental stages. Children are continuously exposed to new pathogens as they explore their environment, attend school, and interact with others. This constant exposure helps their immune system develop and adapt, building a diverse repertoire of immune cells [3]. Children also possess a remarkable ability to generate a robust immune response, often characterized by fever and a rapid increase in specific immune cells, when confronted with infections. Children generally have more resilient immune systems; they may also be susceptible to certain infections due to their immune system’s ongoing maturation [4].

Nutrition plays a vital role in supporting a robust immune system. An adequate intake of essential nutrients, including vitamins (such as vitamins C, D, and E), minerals (such as zinc and selenium), and other bioactive compounds, is essential for maintaining immune function. Deficiencies in these nutrients can compromise immune response and increase susceptibility to infections [5]. Certain dietary components, such as antioxidants and anti-inflammatory compounds found in fruits, vegetables, and other plant-based foods, can positively influence immune function. Probiotics, which are beneficial bacteria found in fermented foods, like yogurt, may also contribute to a healthy gut microbiota, supporting immune health [6]. The immune system is a remarkable defense mechanism that relies on a delicate balance of various components and is highly influenced by dietary factors. A mindful approach to nutrition, ensuring a well-rounded and nutrient-rich diet, is instrumental to promoting optimal immune health and overall well-being [7]. In this review, the purpose is to elaborate on the relationship between malnutrition and the immune system, and how this might relate to infections in children, as well as to describe novel nutritional strategies that can promote an improved health status in these children.

### 1.2. Malnutrition

#### 1.2.1. Undernourishment

Undernourishment occurs when the body lacks essential nutrients, impacting growth and body functions. The causes of this include an inadequate diet, poor absorption of nutrients, economic factors, and illnesses. Undernourishment weakens the immune system, reducing its ability to defend the body against infections, targeting different pathways. The risk factors include poverty, limited food access, poor dietary habits, health conditions, and a lack of education [8]. According to the World Health Organization (WHO), childhood malnutrition is a major risk for their morbidity and mortality, being one of the main causes of the global burden of disease, disability, and mortality among children [9]. More than two-thirds of these deaths globally occur in infants, during their first year of life. Wubante et al. [10] found that the deprivation of colostrum (adjusted odds ratio (AOR): 1.76; 95% confidence internal (CI): 1.01 ± 1.06) and inadequate methods of complementary feeding due to nutritional information gaps (AOR: 2.82; 95% CI: 1.33 ± 5.99) were significant predictors of malnutrition.

Malnourishment among children is a global challenge that extends beyond the simple absence of food to encompass a complex interplay of inadequate nutrition, poverty, and societal disparities [11]. Defined by an insufficient intake of essential nutrients, malnourishment impairs a child’s physical and cognitive development, leaving lasting impacts on their overall well-being. In many regions, factors, such as food insecurity, limited access to nutritious meals, and inadequate healthcare, exacerbate the prevalence of malnourishment among children. Malnourished children are more susceptible to infections, have reduced cognitive abilities, and often face higher mortality rates. The consequences extend far beyond the individual, affecting communities and societies at large, hindering economic development and perpetuating cycles of poverty [12]. Addressing childhood malnourishment demands a multifaceted approach that combines nutritional interventions, access to clean water, healthcare, and educational programs. Global initiatives aimed at eradicating hunger and promoting sustainable agriculture play a crucial role, as do community-based efforts to raise awareness and provide support to vulnerable families. By prioritizing the nutritional needs of children and addressing the root causes of malnourishment, societies can break the cycle of poverty, empower future generations, and foster a world where every child has the opportunity to thrive and reach their full potential.

The WHO is undertaking a new guideline for the prevention and management of undernourishment. This global action plan to fight acute malnutrition in children under the age of 5 years old is based on a more comprehensive approach, calling for a greater integration of nutrition services into healthcare systems and to strengthen those healthcare systems [13]. It is a child-centered approach, with critical importance in breastfeeding and nutrient-dense home diets. It includes specific protocols for milk supplementation, rehydration, hydrolyzed formulas, or ready-to-use therapeutic food in order to properly treat malnourished and/or nutritional edemas in children under the age of 5 years old [14].

Although breastfeeding is the best strategy to lower infant morbidity and mortality, it should be reconsidered in the contexts of undernourishment and infections where maternal diets are inadequate. In these environments, breastfeeding may result in suboptimal lactation unless maternal nutrition is supported [15].

#### 1.2.2. Overweight and Obesity

The other type of “malnutrition”, where children are over-nourished, is presented as being overweight and obese. Being overweight and obese, characterized by excess body weight, stem from factors, like a poor diet (e.g., excessive consumption of foods that lack nutrition, such as sugar), lack of exercise, genetics, and certain medical conditions. These conditions contribute to chronic inflammation, impacting immune function. Higher body mass indices (BMIs) and abdominal fat increase susceptibility to infections and alter the body’s immune response, potentially reducing the effectiveness of vaccines. Lifestyle factors, including sedentary habits and unhealthy diets, pose significant risks [8]. Childhood obesity is a growing public health concern that has reached epidemic proportions globally. Defined as an excessive accumulation of body fat, obesity in children is a multifaceted issue with far-reaching consequences. The rise in sedentary lifestyles, increased consumption of high-calorie low-nutrient foods, and a decline in physical activity are primary contributors to this alarming trend [16]. Beyond the physical ramifications, children grappling with obesity often face social and psychological challenges, including bullying, a low self-esteem, and a higher risk of developing mental health disorders [17]. The long-term health implications are profound, as obese children are more likely to carry their weight issues into adulthood, predisposing them to a myriad of chronic conditions, such as diabetes, cardiovascular diseases, and orthopedic problems. Additionally, obesity in childhood can impact their academic performance, as it may hinder cognitive development and concentration. Addressing this epidemic requires a comprehensive approach, encompassing not only individual behavior changes but also community-wide initiatives, school programs, and policy interventions. Encouraging healthy eating habits, promoting physical activity, and fostering a supportive environment for children to make healthier choices are crucial steps. Families, schools, healthcare providers, and policymakers play pivotal roles in creating a culture that prioritizes the well-being of our youngest generation. By implementing preventive measures and offering resources for intervention purposes, we can mitigate the long-term effects of childhood obesity, fostering a healthier future for our children and breaking the cycle of this pervasive health crisis [18].

It should be mentioned that undernourished children, such as those with a low birth weight, those displaying stunting, or children exhibiting inappropriate weight gain during their preschool and school years, are known to be at a greater risk of obesity in later life [19]. Thus, in 2017, the WHO created specific guidelines for the assessment and management of infants and children at primary healthcare facilities, primarily for use in low- and medium-resource settings. Some recommendations included not providing supplementary foods to moderately malnourished or stunted infants and children, as well as providing nutrition counseling to caregivers of overweight children aged less than 5 years old [20]. In addition, the WHO is guiding another primary healthcare approach for reducing obesity-associated disabilities in children. It includes children with body function impairments, body structures, and/or activity limitations [21]. It is expected to create a project plan by the end of this year (2023).

### 1.3. Malnutrition Increases the Risk of Infection

The strong synergistic association between malnutrition and infection has been recognized for more than 50 years as originally described [22]. Since then, a significant amount of research has been conducted in this area, and there is a unanimous agreement among the authors that mortality is significantly higher in malnourished children compared to healthy ones. However, a number of important aspects about the impact of malnutrition on host defense mechanism have not been well studied, and very few studies have investigated the impact of nutritional interventions on ameliorating malnutrition-infection synergism. A recent study [23], which involved a sizable number of hospitalized Gambian children, amply demonstrated the connection between increased mortality rates linked to numerous respiratory viral diseases and malnutrition, which is typified by a lower weight relative to age.

*Mycobacterium tuberculosis*, the most common infectious illness that kills people, infects one-third of the world’s population [24]. In developing countries where protein-energy malnutrition is also common, this infection is especially impacted by malnutrition and is a leading cause of morbidity and mortality [25]. Furthermore, the results from experimental models show the importance of malnutrition as a major risk factor for tuberculosis [26]. In a similar way, undernourishment can impact the clinical result of tuberculosis. A recent meta-analysis revealed a link between a higher risk of active tuberculosis and low serum vitamin D levels [27]. It is crucial to emphasize that tuberculosis is a common illness whose course, marked by ongoing inflammatory processes, aggravates malnourishment and results in classic cachexia. IgG1 antibodies have been partially blamed for this, as they increase proinflammatory cytokine levels (IFN-γ and IL-6) but not anti-inflammatory cytokine levels (IL-10) [28]. Co-infection with HIV and inadequately followed treatment regimens have been primarily blamed for the rise of extremely severe drug-resistant strains of *Mycobacterium*. It has been proposed that malnutrition has played a role in the increase in drug-resistant *M. tuberculosis* strains [29].

Even with excellent vaccinations, measles still kills and seriously injures children across the world. This virus can cause complications in practically every organ or system, such as encephalitis, laryngitis, and pneumonia. Malnutrition and vitamin A deficiency are two factors that increase the incidence of problems [30]. There is experimental evidence that children who receive vitamin A supplements have a 23% to 30% lower chance of dying young and a lower severity of illness [31]. Because of this, the WHO advises giving measles-stricken children who live in locations where there is a vitamin A deficiency oral doses of vitamin A or zinc [32].

The geographic distribution of intestinal parasitism and malnutrition is comparable, and the same people can have both conditions at the same time [33]. Malnutrition increases susceptibility to infection, while the infection itself causes malnutrition to become more severe. These two causal pathways explain the coexistence of nematode infection and malnutrition. Due to their ability to generate anorexia and a range of pathophysiological reactions in the gastrointestinal tract, including vomiting, diarrhea, and malabsorption, intestinal nematodes can cause malnutrition [34]. All these changes make it more difficult for the host to receive enough nutrients from food. *Giardia duodenalis, coccidia, Entamoeba histolytica*, soil-transmitted helminths, and *Schistosoma* sp. are among the parasites that blatantly alter nutritional status [35].

Furthermore, there is broad agreement that malnutrition raises human malaria morbidity and death rates [36]. This finding is corroborated by controlled clinical trials showing that zinc or vitamin A supplementation can significantly lower clinical malaria outbreaks [37]. The views on the impact of specific micronutrients—like iron or zinc, for instance—continue to differ [38].

Noma is an opportunistic infection caused by severe poverty that spreads rapidly from inflamed gingiva to disfiguring orofacial gangrene. While it exists all over the world, sub-Saharan Africa is where it is observed far more frequently. It is often preceded by measles, malaria, and severe necrotizing ulcerative gingivitis. It is the consequence of extremely complex interactions between malnutrition, infection, and weakened immunity [39].

The current pandemic caused by the widespread SARS-CoV-19 infection has once again highlighted the risk factors associated with obesity, whose prevalence has risen to 13% worldwide, including a greater susceptibility to infections and the likelihood of a more severe illness course. This connection has not received enough attention to date. It is generally accepted that impaired innate and adaptive immune responses are the main causes of obesity-related susceptibility to infectious diseases. A number of cofactors, including compromised respiratory mechanics, skin and subcutaneous tissue homoeostasis, obesity-related comorbidities, and inadequate antimicrobial medication, can indirectly contribute to the beginning and/or aggravation of infectious diseases. Obese subjects are more likely to experience cutaneous infections, most likely as a result of an altered skin barrier and wound healing processes. Additionally, being overweight is linked to a higher incidence of lower and upper respiratory tract infections, as well as an increased risk of urinary tract infections and their recurrence. Furthermore, it appears that obese individuals who undergo general, orthopedic, gynecological, and bariatric surgeries are more likely to develop surgical site infections. Due to the typically inadequate documentation of anthropometric measurements, the information regarding the various infectious diseases associated with obesity is very sparse. Additionally, there are no specific treatment procedures available for obese people, particularly when it comes to the use of additional vitamins and antibiotic medication [40].

## 2. Methodology

The bibliographic search was conducted in November 2023. The literature review was performed as a comprehensive review; thus, it did not follow the systematic review guidelines. The databases employed were the Web of Science and Scopus. The keywords employed were ((malnutrition OR undernourishment OR overweight OR obesity) AND “immune system”) AND (“oral administration” OR “ingestion” OR “intervention”). No filters considering the year of publication were applied, but recent articles were prioritized in the selection of articles. As no standardization of the methodology could be found, the selection of articles was agreed on by two independent authors aiming to provide the reader with a full overview of the current literature available (in terms of the methodology and type of study).

## 3. Immune Systems of Malnourished Children

### 3.1. Undernourishment

#### 3.1.1. Vitamins and Minerals

Childhood malnutrition affects innate and adaptive immune functions, reducing the ability of the immune system to produce an appropriate immune response against infections. Micronutrients are essential for the regulation of immune system function, especially zinc and vitamin A. Vitamin A is an essential micronutrient that maintains proper physiological well-being. It is not synthesized in our bodies and, subsequently, it should come from the diet. However, not all people are able to achieve this. At least 250 million children living in developing countries suffer from hypovitaminosis A, according to the WHO [41]. Additionally, HIV/AIDS is an underlying factor of malnutrition in children [42]. There is a malnutrition prevalence rate of 40–64% in children with HIV/AIDS [43]. A cross-sectional study conducted in Ghana demonstrated that undernourishment was higher in children with HIV/AIDS, suffering inadequate caloric and micronutrient intakes, particularly folate, vitamin C, and iron deficiencies [44].

Vitamin A is associated with higher CD4 and Tβ4 concentrations in children aged from 3 to 17 years old with sufficient vitamin A levels compared to a vitamin A-deficient group in a cross-sectional study performed in Egypt [45]. A Nigerian study [46] significantly associated undernourishment and Zn deficiency with pneumonia. Hypovitaminosis A and low serum Zn were also associated with pneumonia severity. They also demonstrated that Zn deficiency was an independent predictor of pneumonia in children aged from 2 months to 14 years old. In addition, primal studies [47] found an association of Zn deficiency with depressed cell-mediated immunity in protein-energy malnourished children.

#### 3.1.2. Proteins and Fatty Acids

Proteins play a vital role in supporting the immune system, and a deficiency can negatively impact immune function. Antibody production, crucial for adaptive immunity, may be compromised, leading to a weakened response against pathogens. Proteins are essential for the proper functioning of immune cells, including T and B cells, and influence the production of cytokines that regulate immune responses. The complement system, involved in inflammation and pathogen destruction, also requires sufficient protein. Moreover, proteins contribute to wound healing, and their deficiency can impair tissue repair. On the other hand, dietary fats, particularly essential fatty acids, like omega-3 and omega-6, are integral to immune system function. Fats contribute to the structure of cell membranes, aiding immune cells to recognize and respond to pathogens. Omega-3 fatty acids, with anti-inflammatory properties, help regulate the immune response, and their deficiency may lead to imbalanced reactions and chronic inflammation. Fats are essential for antibody production, phagocytosis, and the absorption of fat-soluble vitamins crucial for immune health. A deficiency in dietary fats can compromise these processes, impairing the body’s ability to fight infections and maintain its overall immune function [48].

#### 3.1.3. Amino Acids

Considering the constituents of proteins, amino acids play a crucial role in supporting various functions in the body, including the proper functioning of the immune system. The immune system relies on a balanced and adequate supply of nutrients, including amino acids, to function optimally. For instance, arginine is important for the function of immune cells, including T cells and macrophages; thus, its deficiency may cause impaired immune responses and a decreased ability to combat infections, similar to lysine or tryptophan. In addition, histidine, being a precursor to histamine, which is involved in inflammation and immune responses, is important as a deficiency of this element may affect the body’s ability to mount an appropriate inflammatory response to infections. In the same line, tryptophan is a precursor to serotonin and cysteine is a precursor to glutathione, molecules involved in regulating immune response, and consequently, their absence may result in a more vulnerable immune system [49].

#### 3.1.4. Stunting

Stunting emerges in the early infancy stage and is associated with a dysfunctional immune system, increasing the risk of mortality in children below the age of 5 years old. Here, the child does not achieve their linear growth potential due to chronic or recurrent undernourishment. It affects 20% of children under the age of 5 years old worldwide, contributing to 45% of deaths. In Bangladesh, Kupkova et al. [50] discovered that there were two global changes in the epigenomes of stunted children. One was H3K27ac, which occurs during the first year of life and is associated with probable initial hyperactive immune responses followed by a reduced metabolic capacity in children, suggesting that the limitation of one-carbon metabolites may play a key role in the development of stunting. Moreover, they also observed high HeK9me3 levels in infants between birth and one year of age with poor linear growth results [51]. They suggested that these levels were elevated through the suppression of transposable elements involved in the development of immune responses to pathogens. This pattern can be a predictor of the risk of stunting. It also supports a model where the immune system of stunted children becomes compromised in early infancy.

#### 3.1.5. HIV

A clinical trial conducted in Tanzania enrolling HIV-exposed (but uninfected) infants ingesting multivitamin supplements (vitamins B complex, C, and E) demonstrated that stunted infants had significantly lower measle Ig concentrations than non-stunted infants [52].

### 3.2. Overweightedness and Obesity

#### 3.2.1. Allergic Diseases

Malnutrition has been linked to allergic disorders in children. Allergic disorders are mediated by Th2 responses. A study performed in Indonesia linked being overweight with allergic sensitization (Skin Prick Test reactivity, OR 2.68, 95% CI: 1.50–4.78; Leptin, GMR 3.55 95% CI: 2.99–4.23, and IGF-1, GMR 1.45, 95% CI: 1.15–1.82) in children [53]. A persistent high body mass index (BMI) is also associated with an increased risk of doctor-diagnosed asthma among children, both in infancy and at a school age (OR: 2.9, 95% CI: 1.3–6.4), as well as with an increased risk of atopic asthma (OR: 4.7, 95% CI: 2.0–11.0) [54]. Prenatal obesity can cause changes in the immunophenotypes of offspring [55]. After 10 years of follow-up sessions, Zhang et al. [56] demonstrated that higher prenatal BMIs were significantly associated with the development of allergic diseases in children (HR: 2.45, 95% CI: 1.08–5.57).

#### 3.2.2. Metabolic Syndrome

The excessive consumption of added sugars has indirect but potentially negative effects on the immune system. A high sugar intake is associated with chronic inflammation, obesity, nutrient imbalances, insulin resistance, and disruptions in the gut microbiota, all of which can compromise immune function [57].

Being overweight is the main risk factor for pediatric hypertension. An activated immune system is involved in the origin and progression of both diseases. A study conducted in Germany determined the transcriptional activation of cytotoxic genes (GZMB, PRF1) and proinflammatory mediators (IL-1β, FOS) in adolescents with pediatric hypertension [58]. These factors can increase the risk of long-term effects in these individuals, underscoring the need to monitor hypertension in children in order to prevent adverse long-term cardiovascular events. Carbohydrate beverages consumed by children who are obese or overweight may induce an inflammatory response, also linked to long-term cardiometabolic comorbidities [59]. In a clinical trial that administered two different beverages (carbohydrate and high-protein drinks) to healthy and obese/overweight children discovered that inflammatory monocytes were lower after consuming the carbohydrate beverage, particularly in obese or overweight children [60]. In addition, the increase in blood glucose levels after the consumption of the carbohydrate beverage was associated with a decrease in monocyte populations. These findings suggest that high-carbohydrate meals contribute to underlying inflammatory diseases associated with being obese or overweight.

#### 3.2.3. Physical Activity

**Figure 1:**
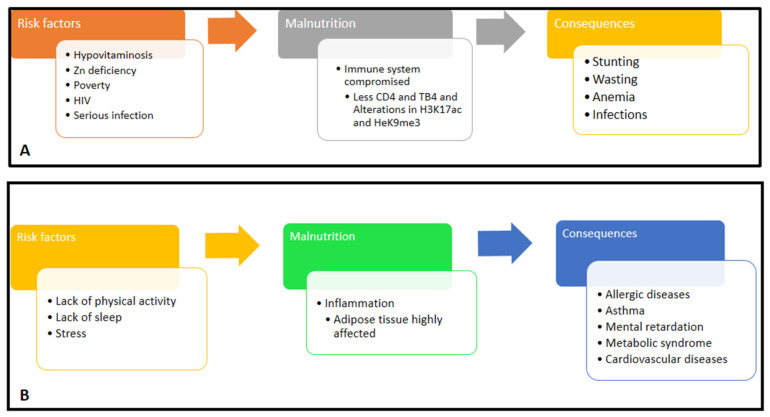
(**A**) Relationships between risk factors, immune system, and consequences of undernourishment. (**B**) Relationships between risk factors, immune system, and consequences of being overweight/obese.

The primary treatment for preventing childhood obesity is lifestyle modification, including physical activity [61]. Exercising has beneficial effects on the symptoms of metabolic syndrome and low-grade systemic inflammation in obese children [62]. The potential therapeutic effect of physical activity on low-grade systemic inflammation was demonstrated in an Italian study, where significant reductions in neutrophils, C-reactive protein, IL-6, IL-8, IL-9, human interferon-inducible protein 10, the granulocyte-macrophage colony-stimulating factor, platelet-derived growth factor subunit b, and eotaxins were observed in physically active obese children compared to sedentary ones. Sleep is essential to support the functions and health of the body, such as immune system activity. Bonnano et al. [63] highlights that children who sleep less are at risk of being obese and/or overweight, due to dysfunctional eating behaviors and decreased physical activity, which cause metabolic changes. In Figure 1, the relationships between risk factors, the immune system, and the consequences of malnutrition, both undernourishment (1A) and overnourishment (1B), are indicated.

Another study that demonstrated how obesity could trigger inflammation and neuroinflammation at a pediatric age was a case report presented by Zingale et al. [64]. They analyzed adipose tissues from obese and normal-weight children, observing alterations in the lipid and fatty acid metabolism levels in obese children, with an onset of inflammatory responses them presented by the genes involved in neuroinflammation. GRN and SMO were upregulated, while IFNGR1 and SNCA were downregulated.

## 4. Nutritional Interventions’ Effect on the Immune System

### 4.1. Undernourishment

#### 4.1.1. Protein Supplementation

Immunity, both humoral and cell-mediated, is severely compromised in protein-energy malnutrition cases, but may be restored with a proper diet. Because maternal starvation causes epigenetic alterations in kids, it is becoming more and more evident that malnutrition can affect immunological development prior to conception. It has been determined that the first 1000 days of life, or from conception to two years of age, represent a developmental window of opportunity for malnutrition treatment interventions as well as a crucial time for immunological development [65]. Ready-to-use therapeutic foods are solid meals created by modifying the formula of the F-100 liquid diet, which is, at present, the WHO recommended diet for children experiencing the rapid catch-up treatment of medical care for severe acute malnutrition. The finished products were quite successful in promoting weight gain in adults and children who were severely and moderately malnourished [66]. Additionally, different authors have assessed the effects of specific nutrients on the immune system to evaluate whether a specific response can be achieved and to understand how nutrients modify the immunometabolic status of children.

A pilot study on the effects of curd and leaf protein concentrates in children with protein energy malnutrition reported improvements in the immune system [67]. The purpose of this study was to determine how the supplementation could affect the nutritional status and immunity of malnourished children (*n* = 80, mean age: 24.91 ± 11.13 months) including protein-energy malnutrition, as measured by anthropometry, hemoglobin, ferritin levels, T-cell subpopulation, and C-reactive protein. The intervention led to increases in weight, hemoglobin level, and CD4:CD8 T-cell subpopulation, and a decrease in serum ferritin. Although confirmation using a larger population is needed, these authors indicate the beneficial effects that these ingredients might have on the immune systems of malnourished children, hastening immune recovery.

Similarly, the immune responses of severe malnourished children treated according to the protocol of the WHO were assessed [68]. In this study, a comparison of the innate immune system of severely malnourished children compared to healthy children was performed. The group of children (*n* = 20, 10 severely malnourished, 10 healthy children, younger than 2 years old) were compared and the malnourished group started the WHO’s nutritional rehabilitation protocol. Several parameters were evaluated, from which the main relevant results were that the malnourished group displayed better phagocytic functions, release of oxygen radicals, and reductions in the number of lymphocytes after the intervention for 30 days. In comparison to the healthy children, the malnourished group had lower lymphocyte values and a lower production of free radicals. Further studies should evaluate whether longer durations of nutritional interventions can completely counteract the immunodeficiency of patients to achieve the immune profile of the healthy children.

#### 4.1.2. Probiotics

*Lactobacillus* plays an essential role in the immunomodulation of the intestinal mucosa as a probiotic bacterium. Probiotics present health benefits, especially in diarrhea diseases, which are one of the main causes of malnutrition. The impact of heat-killed *Lactobacillus casei* on the immune function of macrophages in malnourished children was evaluated by Rocha-Ramirez et al. [69]. In this study, the authors determined the in vitro effects on monocyte-derived macrophages (MDMs) from malnourished infected children and compared them with those of well-nourished healthy children and well-nourished infected children. The results showed an increase in secretion cytokines (TNFα, IL-1β, IL-6, and IL-10 levels), phagocytosis and bactericidal activity, suggesting the potential of this bacteria to modulate the immune, function of macrophages. A clinical trial conducted in children younger than 8 years old in Ethiopia demonstrated that the consumption of *Lactobacillus acidophilus* increased immune cell levels in malnourished children, especially in the age group of 7–8 years old [70]. After 35–40 days of *Lactobacillus* feeding, a dose of at least twice a month was enough to overcome lactose intolerance and diarrhea in these children.

#### 4.1.3. Micronutrient Supplementation

Recently, Noor et al. [71] indicated the effect of changes in the dietary patterns on the immune cells of undernourished children. For three months, the children (*n* = 90, aged between 12 and 18 months old) ingested a micronutrient supplement daily for 6 days a week, one egg, and 150 mL of cow’s milk. At the end of the intervention, peripheral blood mononuclear cells (PBMCs) were isolated to evaluate the phenotypic profiles for CD3+cells, CD4+cells, CD8+cells, NKT cells, and B cells, which were did not to change after the intervention. Nonetheless, activated B cells (CD25+) increased, as well as several pro-inflammatory cytokines, including IL-6, IFNγ, and TNFα.

Community-acquired pneumonia (CAP) is one of the leading causes of mortality in children under 5 years old. The mortality rate is much higher in developing countries due to malnutrition and micronutrient deficiencies that weaken the immune system. Said et al. [32] performed a clinical trial where they demonstrated that supplementations with zinc or vitamin A in children aged 6 months to 5 years old reduced the length of hospital stay and the duration of pneumonic effusion in children with CAP.

Supplementations with folate, vitamin C, and iron are recommended by Intiful et al. [44] in children with HIV/AIDS, where there is a higher prevalence of malnutrition. These children present a weaker immune system, increasing their risk for recurrent infections, such as diarrhea and tuberculosis. The clinical trial, presented above, of multivitamin supplementation (vitamins B complex, C, and E) in HIV-exposed (non-infected) Tanzanian infants did not improve their measles vaccine responses. However, they observed reduced vaccine responses in short infants, as previously mentioned. Therefore, studies are needed to better understand the effects of undernourishment and nutrition supplementation on vaccine responses [50]. There are many immunization and nutritional intervention programs planned for children in sub-Saharan Africa, but there remain few published data on their interactions.

As previously mentioned, vitamin A is an important micronutrient for the proper activity and maintenance of the immune system. Vitamin A supplementation increased CD4 and CD8 T-cell counts in children with concomitant hypovitaminosis A and anemia in Brazil [41]. It also increased CD4 T cells in low-birthweight Bangladeshi infants [72], as well as amplified the expression of CCR9, a retinoic acid-sensitive gene product involved in the regulation of the intestinal mucosa, decreasing intestinal inflammation. These results suggest that vitamin A supplementation at birth has sustained benefits for low-birthweight infants, perhaps by improving their intestinal immune function leading to a better absorption of nutrients. In fact, as already mentioned, it was demonstrated that children who received vitamin A supplements showed a reduction of up to 30% of their chances of dying young and a lower severity of illness [30], or also that supplementations with zinc or vitamin A were associated with reduced clinical malaria outbreaks [37].

Theophilus et al. [73] suggested the need to consume fresh food in order to ensure an adequate dietary intake of calcium, iron, and zinc. For this, in low-income populations, we should incorporate nutrition education and locally available food options, making these foods and supplements affordable and accessible.

### 4.2. Overweight and Obesity

As previously described, obesity exerts negative effects on the components of the immune system and its function. The immunological changes that occur in obese children affect both humoral, especially the secretion of antibodies, and cellular immunity. Obesity is associated with low-grade inflammation, which can lead to the development of different diseases.

#### 4.2.1. Protein Supplementation

Roth et al. [74] evaluated how a change in lifestyle (targeted reductions in fat and sugar intake) of obese children could impact cardiovascular risk factors, insulin resistance, and inflammatory markers, and how the changes are related. For this purpose, obese children (*n* = 115, average age of 10.7 years old), who were compared to normal-weight children as the control, were evaluated. Only 62 could be followed during the intervention for one year. According to the authors, the correlations between the BMI and resistin as well as monocyte chemoattractant protein–1 were evident. Adiponectin was discovered to present significant relationships with many inflammatory mediators, including TNFα and IL-1β, IL-6, and IL-8. Similarly, variations in IL-1β were positively linked with variations in IFNγ, IL-6, IL-8, TNFα, and weight status. This report showed a substantial correlation between several metabolic risk variables before and after weight changes and the immunometabolic status of children. Similarly, Garanty-Bogacka et al. [75] reported that changes in lifestyle (6-month program of hypocaloric diet together with moderate physical activity) in children with obesity (*n* = 50, average age: 14.2 years old) led to health status improvements. These authors evaluated the loss of weight, as well as serum levels of inflammatory markers, including high-sensitive C-reactive protein, IL-6, fibrinogen, white blood count, glucose, insulin, insulin resistance index (HOMA IR), glycosylated hemoglobin (HbA1c), lipids, as well as systolic and diastolic blood pressure levels. After the intervention, the authors indicated an average weight loss of 5.3 ± 3.4 kg, while regarding the immunometabolic status, decreases of serum IL-6, C-reactive protein, white blood count, and fibrinogen were observed, which were statistically correlated with the changes in body fat and HOMA-IR outcomes. The authors also indicated that additional investigations were needed to determine if this reported relationship was causally linked to an improvement in insulin resistance and could help to avoid problems associated with obesity.

A study conducted in Mexico evaluated the colostrum of mothers with obesity, observing a reduction in B cells and considerable cell-specific phenotypic alterations in all leukocyte subtypes [76]. These alterations included the regulation of cell size, internal complexity, and surface expressions of CD45 and CD16, with higher neutrophil average proportions (1.5- to 5-times higher). This pioneering study is a steppingstone to investigate the consequences of these alterations in colostrum on the children that are nourished with it.

#### 4.2.2. Probiotics

Kelishadi et al. [77], considering the relationship between the gut microbiota and immune system, evaluated the anti-inflammatory effects of a symbiotic supplement (viable *Lactobacilli* of human origin and fructo-oligosaccharides) on overweight and obese children and adolescents. The design of the study was a randomized triple-masked controlled trial in which the population (*n* = 70, aged from 6 to 18 years olde) ingested the test item for eight weeks. According to the authors, the supplementation led to a decrease in TNFα and IL-6 levels, together with an increase in adiponectin, although these changes were dependent on the weight reduction.

Recently, the improvement of inflammatory status following a supplementation with *Bifidobacterium pseudocatenulatum* CECT 7765 in insulin-resistant obese children was reported [78]. In this study, obese children (*n* = 48 obese, aged from 10 to 15 years old) received capsules with or without probiotics (10^9–10^ CFU) daily for 13 weeks. After the intervention, circulating high-sensitive C-reactive protein and monocyte chemoattractant protein-1 were significantly reduced, as well as the changes in the microbiota.

#### 4.2.3. Vaccines

Obese children are also less responsive to vaccines than regular-weight ones. Current vaccine adjuvants are less effective at stimulating immune responses in children compared with adults. Brennan et al. [79] identified cytosolic nucleic acids as novel candidate adjuvants for childhood vaccines, demonstrating that obese children and neonates born to obese mothers present functional cytokine responses to the cytosolic nucleic acid Poly(I:C).

Overall, in the case of obese children needing to improve their health status, the dietary considerations include limiting the number of sugar-sweetened drinks and meals that include portions of fruits and vegetables consumed, avoiding fast food and promoting small serving sizes [80].

## 5. Gaps and Perspectives

The effect of nutritional interventions on the immune system of malnourished and obese children is a topic of interest. However, in spite of the promising results defended in this study and retrieved from different reports, there are many limitations in this research field that might hinder an adequate progression of the topic, and that needs to be overcome in order to have reliable scientific evidence to promote regulatory measures for creating a healthier society. Malnutrition influences the immune system through epigenetic changes, affecting gene expression. In addition, T-cell function is compromised, reducing the body’s ability to combat pathogens. Additionally, malnutrition disrupts the balance of the gut microbiota, leading to dysbiosis, which hampers the immune-regulatory functions in the gut. These interconnected effects contribute to immunocompromizing outcomes, increasing individuals’ vulnerability to infections and health issues.

One of the main limitations observed in the studies was the definition of the groups, as sometimes a the “healthy” children group was not included, which should have also been included as the control to measure how the different variables could fluctuate based on the intake of specific nutrients [71]. Regarding the nutritional interventions, it is complicated to homogenize them in different children and to properly register the real intake and how the different macro- and micro-nutrients might impact the development of the subjects and the impact the immune system. In this sense, usually, nutritional interventions do not consider the dose–response effect, which can help us to understand the specific nutrients that promote changes in the immune system. Similarly, the number of parameters that can be measured is usually limited in the studies, hindering the possibility to establish relationships between them, which may explain the pathways involved in the immune response. In this regard, the inclusion of specific parameters is of interest, including CD4/CD8 and CD3/CD19 ratios, as these factors are considered as the best immune biomarkers of nutritional status, as they are modulated in situations of malnutrition [81].

Regarding the various policies, in the European context, for the authorized health claims relating to the development and health of children, vitamin D is included to contribute to the proper functioning of children’s immune systems. A follow-up session on how the research can be translated into official guidelines to ameliorate children’s immune systems is also relevant to create a bridge between the clinical research and communication and labeling of health claims for consumers.

The effects of nutritional interventions on immune function and other parameters (especially body weight) in malnourished adults has also been evaluated in many studies [82,83]. However, the differences in the physiology and metabolism between children and adults must be noted and taken into account in the extrapolation of the results or for the understanding of the underlying mechanisms of how specific nutrients affect the immune systems in target populations.

According to Rytter et al. [84], 245 reports were published from 1957 and 2014 characterizing the immunometabolic status of undernourished children (aged 0–5 years old). However, it must be noted that the techniques employed decades ago are presently considered outdated and their relevance might not be sufficient, compared to recent studies, considering that the tendency in the current research is the harmonization of techniques and the adherence to international guidelines. In fact, the use of serum biomarkers for identifying or tracking malnutrition is debatable, especially in light of the more recent findings. Their limited specificity and the significant impact of underlying conditions, like inflammation, especially on serum visceral proteins, account for this. Furthermore, extensive randomized controlled studies have not been conducted to examine the function of biomarkers in directing nutritional treatment [85].

Future, well-planned, prospective cohort studies should investigate the relationship between immunological markers and morbidity and mortality in children who are malnourished, including providing specific details for nutritional status—ideally including body composition—infections, enteropathy, and low-grade inflammation. Immune markers should be included as outcomes when evaluating dietary and therapeutic therapies for malnutrition [84]. In fact, the innovative experimental methods used to investigate the gut microbiota, virome, immunometabolomics, immunological, and nutritional sensing factors also provide novel chances for the application of interventions to the immunological research on malnutrition in children [65].

Overall, it must be taken into account that, as children grow, their immune system gradually becomes more sophisticated and regulated, resembling the mature immune system of adults. Understanding the unique features of the pediatric immune system is essential for promoting overall health and designing age-appropriate strategies for disease prevention and management [4]. Although we know about the benefits of breastfeeding and the lack of optimal lactation by malnourished women in developing countries, no maternal nutrition support interventions have been found in the literature. Christian et al. [15] suggested that the absence of global policies for the nutritional support of the postpartum mothers could be due to a lack of scientific evidence in the field. Therefore, the scientific community should be encouraged to create nutritional interventions for these malnourished mothers in developing countries.

## 6. Conclusions

Overweight and obesity are increasing at an alarming rate all over the world and undernourishment has slowly been increasing in developing countries for several years until the present day. Malnutrition, overweight, and obesity in children have negative effects on their health, growth, and development. Therefore, addressing malnutrition (including overweight and obesity) is important to improve children’s development and well-being, supporting the social development of families and communities worldwide.

In low- and middle-income countries, childhood malnutrition is a common occurrence. In this scenario, the first two years of life are crucial for achieving appropriate physical and mental development. Stunting, wasting, and severe acute malnutrition are the most harmful types of malnutrition in children. On the other hand, the other type of malnutrition refers to the opposite situation, where the ingestion of food is excessive and mostly unhealthy. In both cases, the immune system is compromised, leading to a modulation of the human body that is different to healthy subjects, and the likelihood of developing diseases is higher, including infections. In fact, both innate and adaptive immunity are damaged by malnutrition. This condition can manifest as malnourishment, where children exhibit stunted growth and compromised immune systems, or as overnutrition, contributing to the rise of obesity and related health issues.

The reciprocal relationship between the immune system and malnutrition has been described in the way that immune dysfunction can be considered both a cause and a consequence of malnutrition. In this regard, malnourishment and obesity-related problems can be prevented and managed by even modest weight loss achieved by dietary and activity interventions. In summary, the immune system is a crucial player in the body’s defense mechanisms against pathogens and abnormal cells. Proper nutrition, particularly a well-balanced and nutrient-rich diet, plays a pivotal role in supporting immune function and maintaining overall health, highlighting the interconnectedness between food and the immune system.

## Data Availability

No new data were created or analyzed in this study. Data sharing is not applicable to this article.
